# Epidemiology and Control of Leishmaniasis in the Former USSR: A Review Article

**Published:** 2018

**Authors:** Vladimir SERGIEV, Anatoly KONDRASHIN, Sergei LITVINOV, Lola MOROZOVA, Natalia TURBABINA, Ekaterina STEPANOVA, Maria MAKSIMOVA, Sergei SHEVCHENKO, Evgeny MOROZOV

**Affiliations:** 1. Dept. of Tropical Medicine and Parasitic Diseases, Sechenov University, Moscow, Russia; 2. Institute of Medical Parasitology, Tropical and Vector-Borne Diseases, Sechenov University, Moscow, Russia; 3. Russian Medical Academy of Continuous Professional Education, Moscow, Russia

**Keywords:** Anthroponotic cutaneous leishmaniasis, Visceral leishmaniasis, Zoonotic cutaneous leishmaniasis, Epidemiology

## Abstract

**Background:**

All types of the Old World’s leishmaniasis were endemic on the territory of the South regions of ex-USSR. Epidemiological situation was well under control during the USSR era, due to implementation of complex anti-leismaniasis measures. These interventions were dramatically stopped as a result of the collapse of the USSR.

**Methods:**

Most relevant publications on epidemiology and control of leishmaniases in the Republics of Central Asia and Transcaucasia of the ex-USSR were screened.

**Results:**

Within the endemic area, the foci of different kinds of leishmaniasis are often overlapped thus calling for deployment of integrated measures. The anthroponotic cutaneous leishmaniasis (ACL) was reported in settlements and towns of Central Asia and Transcaucasia of the ex-USSR. The natural foci of cutaneous leishmaniasis were widespread in the desert of Turkmenistan, Uzbekistan, Kazakhstan and Tajikistan. The northern boundary of the zoonotic cutaneous leishmaniasis (ZCL) area coincided with the northern boundary of the distribution of great gerbils – the main reservoir of this infection in the ex-USSR. Visceral leishmaniasis (VL) occurred in the Central Asian Republics and in the republics of the Transcaucasia. Holistic approach was adopted by the programs targeting the source of infection, vector(s) and man.

**Conclusion:**

The presence rise in the number of cases of different types of leishmaniasis in the ex-USSR strongly necessitates that health authorities should consider these diseases as an important public health problem. The immediate task would be rebuilding a comprehensive surveillance system consisting of active and passive case detection mechanism along with immediate treatment of the patients.

## Introduction

All three types the Old World’s leishmaniasis – zoonotic and anthroponotic cutaneous, and visceral – were endemic on the territory of the southern Republics of the USSR in Central Asia and Transcaucasia. Before the collapse of the USSR, full-fledged control of these diseases was carried out in each Republic based on the scientific based knowledge of the epidemiological peculiarities of each disease (1). The result of such interventions was a considerable reduction of prevalence and incidence of all forms of leishmaniasis.

With the disintegration of the USSR, due to various reasons, including nearly the collapse of national health services, accompanied by very slow process of reforms of public health system and services, control and preventive activities in the most of Newly Independent States (NIS) related to infectious diseases have been dramatically decreased. Moreover the staff experienced in the planning, organization, implementation and evaluation of anti leishmniasis activities had to leave the services to earn their living outside health services. Consequently the situation with Leishmaniasis has been deteriorated in all Republics of the southern NIS. In spite of the fact that registration of leishmaniases cases is still compulsory the official statistical information represents the data of passive surveillance only, and the validity of data is often very questionable.

While Tajikistan remains to be an endemic country, visceral leishmaniasis (VL) re-emerged in Azerbaijan, Armenia, Georgia, Kazakhstan and Uzbekistan (2). Official data on VL incidence is considered underreported, while according to the estimates it is 2–4 folds higher than officially reported. In Georgia, for example the annual estimated number of cases varied from a dozen to several hundred cases. Similar situation is with the ACL, which reappeared in Azerbaijan, Georgia, Tajikistan, Turkmenistan, Kazakhstan and Uzbekistan. In the two latter countries the incidence is particularly impressive with annual number of reported cases being 100 cases. Wide spread of ZCL persists in Turkmenistan and Uzbekistan. The disease appeared in Tajikistan and in the southern Kazakhstan (3). It is obvious that the current situation with leishmaniasis in the NIS countries of the former Soviet Union has returned to the level of the beginning of the 20^th^ century due to disruption of sustainable control activities, shortage of experienced medical personnel and the serious problems with drug supply.

In consideration of such a situation, the purpose of this review is to share with the states national authorities the experiences in control and prevention of leishmaniases carried out in the southern republics before the collapse of the USSR. These experiences might serve as a basis for re-establishment of comprehensive leishmaniasis control programs in the ex-Soviet Republics.

## Methods

Only most relevant publications on epidemiology and control of leishmaniases in the Republics of Central Asia and Transcaucasia of the ex-USSR were screened. Unpublished data from the Archives of the Martsinovski Institute of Medical Parasitology and Tropical Medicine were also used for the purposes. Data from foreign publications, especially from countries with similar or close to that epidemiological situation in respect of leishmaniases (e.g. from Islamic Republic of Iran) was also included into the text of an article (4).

## Results

Epidemiological analysis of spatial distribution of leishmaniases in the ex-Soviet Republics in Central Asia and Transcaucasia revealed that the northern borderline of these diseases is determined by the distribution of the vectors (42–46° North latitude). Within the endemic area, the foci of different kinds of leishmaniasis are often overlapped. For example, both anthroponotic and zoonotic cutaneous leishmaniasis occurred in the suburbs of Ashkhabad city (Turkmenistan). Anthroponotic cutaneous and visceral leishmaniasis occurred simultaneously in Samarkand and Tashkent cities (both in Uzbekistan), and Gyandja city in Azerbaijan. At the same time, the ecology and epidemiology of each type of leishmaniasis was specific (5, 6).

The ACL was widespread in settlements and towns of Central Asia and Transcaucasia of the former USSR. Ashkhabad, Tashkent, Kokand in Central Asia, Barda, Gyandja in Azerbaijan, were notorious foci of acl, although sporadic cases of the disease were registered in many other towns as well. The cases were distributed unevently throughout the settlements or towns. Surveys did show that the source of infection lay either in the same courtyard or in a neighbouring one. The microfocal distribution of the new cases was not far from the primary patient due to the short distance of migration of sandflies within the town or settlement (7). The main vector was *P. sergenti,* while *P. papatasi* was found in some foci as well.

After successful control campaigns carried out in 1950s–1960s in the ex-Soviet republics, ZCL caused by *Leishmania major* regained epidemiological significance only until the beginning of the 21^st^ century. Leishmanial infection due to *L. major* was detected in nine species of mammals – *Rhombomys opimus*, *Meriones libycus*, *M. meridianus*, *Spermophilopsis leptodactilus*, *Mus musculus*, *Allactaga severtzovi*, *Mustela nivalis*, *Vormela peregusna*, *and Hemiechinus auritus*. Five other species of mammals – *A. elater*, *Dipus sagitta*, *M. tamariscinus*, *Nesokia indica*, *Hystryx leucura* – were also suspected to be involved in the epizootology of cutaneous leishmaniasis (5, 8, 9). These species of mammals were not equally important as reservoirs of *L. major*. Conditions most favorable for the parasite exist in the skin of the ears of great gerbils. On an average, a lesion (as a rule, a nonulcerated infiltration) persists for 11 months after inoculation in spring by sandflies of the first generation, and 23 months after the inoculation, in July–August, by sandflies of the second generation (10). Thus, parasite survives in great gerbils during an interepizootic (winter) season, and sometimes even during two subsequent seasons following interruption of transmission. Other hosts are less suitable for *L. major* as parasites are extremely scanty in their lesions.

Sandflies are closely linked with gerbil’s burrows. They meet there all the necessary conditions for life and breeding (11). As a rule, other desert animals are considerably less numerous than great gerbils and fail to create conditions suitable for sandflies. Besides *R. opimus*, *M. libycus* only produces numerous holes, thus giving rise to significant numbers of sandflies in some areas (12). At present it is recognized that the following four species of the genus *Phlebotomus* – *P. papatasi*, *P. caucasicus*, *P. andrejevi*, and *P. mongolensis* – play a major part in the dissemination of *L. major* in the NIS countries of the former USSR. Each of these species is the principal vector in the different parts of the enzootic area (1, 13, 14). *R. opimus* is the preferred host for bloodsucking by the genus *Phlebotomus*. Other leishmania hosts (except *H. auritus*) are rarely bitten by sandflies (15).

The natural foci of cutaneous leishmaniasis were wide-spread in the desert of Turkmenistan, Uzbekistan, southern Kazakhstan, and southern Tajikistan. The northern boundary of the ZCL area coincided with the northern boundary of the distribution of great gerbils – the main reservoir of this infection in the former USSR (16, 17). In the eastern part of the Surkhan Darya geographic district, the main reservoir was *M. libycus* (18). The same rodent served as the reservoir of cutaneous leishmaniasis in the eliminated foci in the south Tajikistan in the 1940s (19, 20) and around the town of Nurek (Tajikistan), were cases were occasionally observed.

The natural foci fall into three groups being determined by the presence of vector(s): monovectoral – with one species only -*P. mongolensis*; oligovectoral – 5–6 species present, with the preponderance of *P. mongolensis* ; and polyvectoral with the presence of 10–12 species among which 3–4 species being most widespread and depending on the type of the soil (21, 22).

In the deserts and semideserts of Central Asia human cases of ZCL are extremely rare in spite of the widespread epizootics in gerbils. Most human cases are due to the infection contracted in oases located in the river valleys and in the foothills plains of the southern Turkmenistan and Uzbekistan, where sufficient numbers of *P. papatasi* (the species responsible for the transmission of *L. major* to man) are present. However, the epidemiological activity of natural foci depends not only on the density of *P. papatasi* but also on the number of biotic and abiotic factors influencing the level of transmission (force of infection).

The ZKL cases among human cases in Uzbekistan have been registered in Surkhandarya oblast (in the south-west areas close to to the Amudarya-river valley), in Kashkadarya oblast (bordering with the desert area of Bukhara, Karakul and Karawlbazar oases) as well as in Navoi oblast (in the northern areas bordering the oasis areas of the desert), in Khorezm oblast (the Amudarya right part of the oasis), in Karakalpakia (alongside the eastern border of the oasis territory), in Syrdarya and Dzhizak oblasts (the southern borders of the Golodnaya and Dzhizak steppes, and also in the southwest part of the Samarkand oblast bordering with the plain desert areas.

In general seasonal as well as long term characteristics of ZCL epidemic manifestations in Uzbekistan corresponded to common regularities for that kind of leishmaniasis. As a rule the first cases of the disease among human beings are registered in the second half of August and September. Periodicity of human infection had been observed as typical for many years. Sharp incidence growth among human beings was registered over the whole or in some adjusted to each other areas. After the incidence rise frequently for 1–3 yr it usually comes a fall. The irrigation effect on the morbidity dynamic is especially manifested at early stages of the irrigation activity of new developing lands. This period is characterized by mosaic development of the irrigated areas and virgin areas inhabited by gerbils. The areas still retain the desert look but hydrothermal soil changes are already occurring, as well as changes in microclimate of the great gerbil’s burrows. As a result the *P. papatasi* population increases. The microclimatic transformations in the great gerbil’s burrows and in the desert areas adjusted to the oases appear to be the main and inevitable reason for epidemiologically active ZCL foci in the irrigated areas of the desert and semidesert territories. The non-immune population of new comers to those areas engaged in irrigation activities and land cultivation work appears to be a contributing factor for epidemic of ZCL.

By mid-1980th irrigation reclamation of new areas had been actually completed. The risk of contracting ZCL within highly populated oases was not high, but people living along oasis area bordering a desert remained under high risk of zoonotic cutaneous leishmaniasis. The ZCL epidemiological situation might be deteriorated due to uncontrolled cultivation of perioasis lands being widely privatized as well as due to lesser or even lack of antileishmaniasis measure owing to absence of financial support as well as disintegration of special state owed antiepidemic centres.

In the former USSR, the VL occurred in the Central Asian Republics (Turkmenia, Uzbekistan, Tajikistan, Kirgizia and southern part of Kazakhstan) and in the three republics of the Transcaucasia (Armenia, Georgia and Azerbaijan). The disease affected mainly children, but the cases among the adults were also occurred from time to time (2, 23, 24). *P. kandelaki* and *P. chinensis* in the Transcaucasia and *P. caucasicus, P. chinensis and P. mongolensis* in Central Asia and Kazakhstan were considered to be the main vector (25, 26, 27, 28) The dogs were primary source of the infection in the settlements and towns, while Jackals (*Canis aureus*) and foxes (*Vulpes vulpes* and *V. corsak*) were the natural carriers of VL in the rural foci in addition to dogs (20, 29, 30), the situation being very close to that in Iran (31–33).

There is a significant fluctuation pattern from year to year in the number of registered VL cases. Sporadic cases during several years might reach the size of epidemic outbreak during particular year with dozens of cases (34).

## Discussion

The strategies of control and prevention of leishmaniases in the ex-USSR were based on good knowledge of epidemiology of infections. Implementation of anti leishmaniasis activities was carried out by the personnel of Basic Health Services and Public Health services in cooperation with research institutions and partners, like ministries of irrigation, agriculture, economic development and alike. Financial support came from republican budget and from allocations from specific ministries/companies – partners in leishmaniasis control. Holistic approach was adopted by the programs targeting the source of infection, vector (s) and man.

The control measures against ACL included case detection and prompt treatment, elimination of vectors in micro foci by insecticide application (35). The latter was achieved through insecticide residual spraying of households by malaria control program. Elimination of infected stray dogs within the framework of rabies control program proved to be quite effective in decreasing/eliminating the risk of contracting infection by man.

It was learnt that the efficiency of different control measures against ZCL depended not only on the potentiality of the method itself, but also on the conditions of its application. The artificial inoculation, so called leishmanization, was an effective method of individual protection against cutaneous leishmaniasis. This approach was especially useful for groups and persons staying temporarily in natural foci where other preventive measures were not used.

The application of artificial inoculation among indigenous residents of cutaneous leishmaniasis foci is not indicated as the disease affects local people during childhood, and after cutaneous lesions are healed a person gains protective immunity and becomes resistant to reinfection.

In 1965–1967, mass prophylactic leishmanization against ZCL was undertaken among military recruits in the highly endemic region of the southern Turkmenistan by scientists from Martsinovsky Institute of medical parasitology and tropical medicine (Moscow). The development of specific lesions was observed in 96–100 per cent of inoculated individuals.

The total number of persons inoculated in 1966–1967 during the campaign in Turkmenistan exceeded 9500. The results revealed that leishmanization with a culture of *L. major*, virulent strain, is a reliable protective method against ZCL. Only one person out of 8242 inoculated with such strains did get ill with typical cutaneous leishmaniasis and two others produced abortive lesions 1–2 mm in diameter. 128 out of 1305 persons inoculated with a strain of low virulence, which were living under the same conditions, as those inoculated with virulent strains, did get ill. In noninoculated group in the same foci, the rate of incidence fluctuated from 45 to 405 per 100 with mean rate 79 (36).

The total number of leishmanized persons in the former Soviet Union exceeded 50 000.

The need for a vaccine (s) against cutaneous leishmaniasis and the population at risk for whom such vaccines should be developed had been expressed on a several occasions by the scientific communities of endemic countries (37). Experiences with leishmanization were undertaking elsewhere, notably in the Islamic Republic of Iran. However, leishmanization (a deliberate infection at a predetermined site on the body) with *L. major* has several limitations for mass application, which were describe in Iran (38).

In considerations of those limitations, another approach could be useful. Method of individual protection was tried in the former USSR. Chemoprophylaxis with pyrimethamine administered at 0.02–0.025 g weekly for the entire transmission season was tested in Turkmenistan in 1980 and in Uzbekistan in 1989. The total number of persons under observation was 2313. The efficacy of the method proved to be very high – only 2 cases of ZCL (0.3%) were registered among 625 persons who received medicine under medical supervision (39).

Experience showed that rodent control in small areas with a high probability of reinvasion of rodents from a surrounding untreated territory is epidemiologically ineffective. On the contrary, rodent control used regularly within the natural limits of the local population of rodents is an effective method for protection of local people against zoonotic cutaneous leishmaniasis.

Intensive reclamation of unused lands in Uzbekistan taking place in the 1960s–1980s gave rise to a significant incidence increase. During that period large scale activities to control great gerbil’s populations on the territory of oases were carried out by special teams funded by construction companies. Spectacular results had been achieved by the destruction of *Rhombomys* borrows through plugging to the depth of 0.5 m.

Another approach used was an elimination of great gerbils through poisoning the rodents with zinc phosphide grain baits. As a result of the carried out activities the great gerbil settlements (systems of burrows) were eliminated completely within the vast irrigated areas of the Golodnaya, Dzhizak, and Karshi steppes as well as in some other irrigated territories of Uzbekistan. Modification of this method was developed in Iran where ZCL is an increasing public health problem in many rural areas of the country. Zinc phosphide was used once a month in May, June, July and September within a 500 m circle of houses in the intervention areas. It appeared that changes in the number of rodent holes over time in the intervention and control villages was statistically significant. There was also significant differences in the incidence of the ZCL between intervention and control villages. It was suggested that rodent control operations using zinc phosphide be done within a 500 m circle of houses once every 2 yr before the beginning of the active season of sandflies (40).

The method for sandfly control proved to be less effective. Indoor residual spraying might be effective only in urban and semi-urban areas. Even three rounds per season the insecticide spraying does not reduce either the density of sandflies or the morbidity in villages (41). All means of individual and collective mechanical protection do not ensure complete protection of people from the attack of sandflies and are epidemiologically ineffective (13). The method of skin application of repellents is unacceptable in protecting people because of too short period of efficiency of repellent in hot climate. Impregnated bed nets are also not effective in ZCL foci. That is why the practical application of zoonotic cutaneous leishmaniasis control in the former USSR depended solely on rodent control and leishmanization.

Significant fluctuation from year to year in the number of registered VL cases is typical from single case to several dozens, while the incidence increase is observed once in several years. It happened for example in the sixties and eighties with 25 – 60 cases observed annually (34).

The control measures in regard to VL included detection and destruction of infected dogs, detection and treatment of human cases of the disease and residual insecticide spraying (9, 42). VL control in the sixties was facilitated by the campaign on rabies control implemented at the same time, which included destruction of homeless dogs (22).

## Conclusion

The presence rise in the number of cases of different types of leishmaniasis in the ex-Soviet republics of Central Asia and Transcaucasia strongly necessitates that health authorities should consider these diseases as an important public health problem. This is particularly so that all the republics by and large had already overcome the dire consequences of disintegration of the USSR, and with the assistance of the world community would be able to resurrect their national programs of control and prevention of leishmaniasis. In doing so, the immediate task would be rebuilding a comprehensive surveillance system consisting of active and passive case detection mechanism along with immediate treatment of the patients. Furthermore, due to the presence of various risk factors ranging from anthropogenic ecological disturbances to natural disasters, it is critical to plan future control strategies based on scientifically sound methods of control and prevention of leishmaniasis, described in this paper.

## References

[1] Strelkova MV Ponirovsky EN Morozov EN A Narrative Review of Visceral Leishmaniasis in Armenia, Azerbaijan, Georgia, Kazakhstan, Kyrgyzstan, Tajikistan, Turkmenistan, Uzbekistan, The Crimean Peninsula and Southern Russia. Parasit Vectors. 2015; 8:330. 26077778 10.1186/s13071-015-0925-z PMC4474452

[2] Zhirenkina EN Ponirovskiĭ EN Strelkova MV The epidemiological features of visceral leishmaniasis, revealed on examination of children by polymerase chain reaction, in the Papsky District, Namangan Region, Uzbekistan. Med Parazitol (Mosk). 2011; (3):37–41. [Article in Russian]. 21936088

[3] Alvar J Vélez ID Bern C Leishmaniasis Worldwide and Global Estimates of Its Incidence. PLoS One. 2012; 7(5):e35671. 22693548 10.1371/journal.pone.0035671 PMC3365071

[4] Gholamrezaei M Mohebali M Hanafi-Bojd AA Ecological Niche Modeling of main reservoir hosts of zoonotic cutaneous leishmaniasis in Iran. Acta Trop. 2016; 160:44–52. 27150212 10.1016/j.actatropica.2016.04.014

[5] Kojevnikov PV Dobrotvorskaya NV Latyshev NI. Studies on cutaneous leishmaniasis. Moscow: Medgiz; 1947 (in Russian).

[6] Rodiakin NF. Problems of Immunity and Specific Profilaxis of Borovsky’s Disease (cutaneous leishmaniasis). Ashkhabad: MOH of the Turcmen SSR; 1957 (in Russian).

[7] Moshkovsky SD Nosina VD. The staining of sandflies as a method for the study of features of its biology and ecology. Med Parazitol. 1933; 2: 407–409 (in Russian).

[8] Dubrovsky YA. Mammals as the reservoir in natural nidi of cutaneous leishmaniasis with special references to great gerbils. Novosibirsk: Nauka; 1974, part 2, p. 202–217.

[9] Petrischeva PA Safyanova VM. Geography of Diseases with Natural Focality in regard to its Prevention. Moscow: Medicina; 1969 (in Russian).

[10] Shishlyaeva-Matova ZS. Duration of cutaneous leishmaniasis in Rhombomys opimus Licht. And its dependence upon the season, when the animals acquirer infection. Med Parazitol. 1966; 35 (1): 85–91 (in Russian). 6012304

[11] Vlassov JP. Le terrier du spermophil (Spermophilopsis leptodactylus Licht.) et de la grosse gerbille (Rhombomys opimus Licht.) comme biotope original dans les environs de le ville d’Ashkhabad, Conference Interrepublicaine sur la Leishmaniose Cutanee et le Probleme des Phlebotomes, Ashkhabad; 1941, p. 74–89.

[12] Artem’ev MM Flerova OA Beliaev AE. Quantitative evaluation of productivity of breeding places of sandflies in nature and villages. Med Parazitol (Mosk). 1972; 41(1):31–5. [Article in Russian]. 4260645

[13] Safyanova VM. Biological Relationship between Blood-Sucking Insects and Pathogens of Human Diseases, Moscow: Medicina; 1967: 246–285 (in Russian).

[14] Safyanova VM. Typification of leishmaniasises nidi on the basis of transmission factors. Parasitilogia. 1974; 8: 336–347 (in Russian).

[15] Strelkova MV Djabarov LN. Unequal role of different mammals as a host for bloodsucking by sandflies. Conference on Medical Parasitilogy, Samarqand 1971, p. 226–228 (in Russian).

[16] Dubrovsky YA. Some data on the spatial structure of area of natural nidality of cutaneous leishmaniasis. In: Research on Medical Geography, Moscow Branch of the USSR Geography Society; 1973, p. 35–37 (in Russian).

[17] Dubrovsky YA. Materials on natural focality of cutaneous leishmaniasis in the USSR subzone of northern deserts. Med Parazitol. 1973; 42 (6): 646–655 (in Russian). 4783461

[18] Latyshev NI Kriukova AP Povalishina TR Cutaneous leshmaniasis in Termez. Conference on Medical Parasitology, Samarqand 1971, p. 178–183 (in Russian).

[19] Gozodova GE Martynova ZI Kondrashin AV The special structure of the cutaneous leishmaniasis distribution area in the Western Pamir. Proceedings of 1-st Inter-repuplican Scientific Conference of the Central Asian Republics and the Kazakh SSR on the problem of “The main parasitic diseases, its prevention and treatment”, Tashkent: Medicina; 1967, p. 20–22 (in Russian).

[20] Latyshev NI Kriukova AP Povalishina TP. Essays on parasitic diseases in Middle Asia. 1. Leishmaniasis in Tadjikistan. Materials on medical geography of Tadjik SSR (Results of expeditions in 1945–1947). Regional and General Problems, Experimental Parasitology and Medical Zoology. Transaction of Gamaleya Institute, 1951; 7: 35–62 (in Russian).

[21] Dergacheva TI Zherikhina II. Distribution patterns of sandflies of the genus *Phlebotomus* in colonies of the great gerbil on the territory of the Karshi Steppe. Med Parazitol (Mosk). 1974; 43(4):423–8. [Article in Russian]. 4279038

[22] Remyannikova TR. Distribution of sandflies in natural foci of cutaneous leishmaniasis in different areas of Turkmenia. Parasitologia. 1970; 4: 418–422 (in Russian).

[23] Maruashvili G.M. Visceral leishmaniasis (Sabchota Sakartvelo). Nauka: Tbilisi; 1968 (in Russian).

[24] Mirzojan N.A. Some data on distribution of visceral leishmaniasis in USSR. Med Parazitol. 1957; 26 (1): 47–50 (in Russian).

[25] Chun-Sun F. Study on visceral leishmaniasis in Kzyl-Orda oblast of the Kazakh SSR. Conference on Leishmaniasis and Sandfly fever; 1962, p. 65–68 (in Russian).

[26] Dursunova SM Karapet’ian AB Ponirovskiĭ EN. Some data on the leishmaniasis in the Turkmen SSR. Med Parazitol (Mosk). 1965; 34(3):303–9. [Article in Russian]. 4223773

[27] Nadjafov AY. Leishmaniasis, its vectors and appropriate control measures applied in Azerbaijan SSR. 8th International Congress on Tropical Medicine and Malaria, Tehran, Iran Abstracts and Reviews. 1968, p. 296–297.

[28] Saladze ID. Sandfly species in Eastern Georgia and some features of biology of *P. chinensis* and *P. kandelaki*. Transactions of Virsaladze Institute of Medical Parasitology and Tropical Medicine. 1963; 4: 155–163.

[29] Lysenko AJ Lubova VV. Epidemiology and geography of the visceral leishmaniasis in USSR. International Symposium on Leishmaniasis Ecology Montpellier, France, 1974.

[30] Maruashvili GM Bardzhadze BG. [On the natural focality of visceral leishmaniasis in the Georgian SSR]. Med Parazitol (Mosk). 1966; 35 (4): 462–3 . 4230863

[31] Karimi A Hanafi-Bojd AA Yaghoobi-Ershadi MR Spatial and temporal distributions of phlebotomines and flies (Diptera: Psychodidae), vectors of leishmaniasis, in Iran. Acta Trop. 2014; 132:131–9. 24462940 10.1016/j.actatropica.2014.01.004

[32] Mohebali M Arzamani K2 Zarei Z Canine Visceral Leishmaniasis in Wild Canines (Fox, Jackal, and Wolf) in Northeastern Iran Using Parasitological, Serological, and Molecular Method. J Arthropod Borne Dis. 2016; 10(4):538–545. 28032106 PMC5186744

[33] Mohebali M. Visceral leishmaniasis in Iran: Review of the Epidemiological and Clinical Features. Iran J Parasitol. 2013; 8(3):348–58. 24454426 PMC3887234

[34] Mezgirieva MF Sabitov EA Lesnikova EV Handbook on Leishmaniasis in Turkmen SSR. MOH: Ashkhabad, 1988 (in Russian).

[35] Nadjafov AY. The status of leishmaniasis incidence and its control activities in Azerbaijan SSR. Med Parazitol. 1966; 35 (4): 463–470 (in Russian). 4230864

[36] Sergiev VP. Control and Prophylaxis of Cutaneous Leishmaniasis in the Middle Asia Republics of the Former USSR. Bull. Soc. Franc. Parasitologie. 1992; 10(2): 183–187.

[37] Modabber F. Experiences with vaccines against cutaneous leishmaniasis: of man and mice. Parasitology. 1989; 98 Suppl:S49–60. 2657601 10.1017/s0031182000072243

[38] Nadim A Javadian E Mohebali M. The experience of leishmanization in I.R. Iran. East Mediterr Health J. 1997; 3: 284–289.

[39] Sergiev VP Shuikina EE. Pyrimethamine chemoprofilaxis for Zoonotic Cutaneous Leishmaniasis caused by *Leishmania major*. 1993; WHO/Leish/93.33, 2p

[40] Ershadi MR Zahraei-Ramazani AR Akhavan AA Rodent control operations against zoonotic leishmaniasis in rural Iran. Ann Saudi Med. 2005; 25(4):309–12. 16212124 10.5144/0256-4947.2005.309 PMC6148012

[41] Artemiev MM Kondrashin AV Jarov AA The effect of DDT spraying on the sandflies population in villages. Proceedings of 3-rd Conference on Leishmaniasis and Other Human-borne Tropical Diseases with Natural Focality in the Middle Asia and Trans-Caucasia, Ashabad, 1969, p. 32–34 (in Russian).

[42] Kovalenko DA Nasyrova RM Ponomareva VI Human and canine visceral leishmaniasis in the Papsky District, Namangan Region, Uzbekistan: seroepidemiological and seroepizootological surveys. Med Parazitol (Mosk). 2011; (3):32–7. [in Russian]. 21936087

